# Targeting of TGF-β-activated protein kinase 1 inhibits chemokine (C-C motif) receptor 7 expression, tumor growth and metastasis in breast cancer

**DOI:** 10.18632/oncotarget.2739

**Published:** 2014-12-10

**Authors:** Hui-Ling Huang, Chi-Hsiang Chiang, Wen-Chun Hung, Ming-Feng Hou

**Affiliations:** ^1^ Institute of Biomedical Sciences, National Sun Yat-Sen University, Kaohsiung 804, Taiwan, Republic of China; ^2^ National Institute of Cancer Research, National Health Research Institutes, Tainan 704, Taiwan, Republic of China; ^3^ Department of Surgery, College of Medicine, Kaohsiung Medical University, Kaohsiung 807, Taiwan, Republic of China; ^4^ Department of Surgery, Kaohsiung Municipal Ta-Tung Hospital, Kaohsiung 801, Taiwan, Republic of China; ^5^ Cancer Center, Kaohsiung Medical University Hospital, Kaohsiung 807, Taiwan, Republic of China

**Keywords:** TGF-β-activated protein kinase 1 (TAK1), TAK1 binding proteins (TABs), chemokine (C-C motif) receptor 7 (CCR7), NF-κB, c-JUN, lymphatic invasion

## Abstract

TGF-β-activated protein kinase 1 (TAK1) is a critical mediator in inflammation, immune response and cancer development. Our previous study demonstrated that activation of TAK1 increases the expression of chemokine (C-C motif) receptor 7 (CCR7) and promotes lymphatic invasion ability of breast cancer cells. However, the expression and association of activated TAK1 and CCR7 in breast tumor tissues is unknown and the therapeutic effect by targeting TAK1 is also unclear. We showed that activated TAK1 (as indicated by phospho-TAK1) and its binding protein TAB1 are strongly expressed in breast tumor tissues (77% and 74% respectively). In addition, increase of phospho-TAK1 or TAB1 is strongly associated with over-expression of CCR7. TAK1 inhibitor 5Z-7-Oxozeaenol (5Z-O) inhibited TAK1 activity, suppressed downstream signaling pathways including p38, IκB kinase (IKK) and c-Jun N-terminal kinase (JNK) and reduced CCR7 expression in metastatic MDA-MB-231 cells. In addition, 5Z-O repressed NF-κB- and c-JUN-mediated transcription of CCR7 gene. Knockdown of TAB1 attenuated CCR7 expression and tumor growth in an orthotopic animal study. More importantly, lymphatic invasion and lung metastasis were suppressed. Collectively, our results demonstrate that constitutive activation of TAK1 is frequently found in human breast cancer and this kinase is a potential therapeutic target for this cancer.

## INTRODUCTION

TGF-β-activated protein kinase 1 (TAK1) was originally identified as a protein kinase which activity is stimulated by TGF-β and bone morphogenetic proteins in a complementation screen [1]. Subsequent studies demonstrated that TAK1 is a key mediator in inflammation and can be activated by many pro-inflammatory cytokines like interleukin-1, tumor necrosis factor-α and Toll-like receptor ligands [2–5]. In addition, TAK1 has been shown to involve in the regulation of innate immunity, kidney fibrosis, cardiac remodeling and tumor pathogenesis [6–11].

In cells, TAK1 forms a multiple protein complex and is bound with its binding partners TAB1, TAB2 and TAB3 [12]. Knockout of TAK1, TAB1 or TAB2 in mice all leads to embryonic lethality indicating its activity is critical for development [13, 14]. TAK1 activity is mainly controlled by post-translation modifications. Phosphorylation of the activation loop of TAK1 induced upon cytokine stimulation increases TAK1 activity [15, 16]. Ubiquitination of TAB2, TAB3 and TAK1 also regulates the activation of TAK1 [17, 18]. Wang et al demonstrated that TAK1 is an ubiquitin-dependent kinase and the tumor necrosis factor receptor-associated factor 6 (TRAF6), together with Ubc13, catalyzes the formation of a Lys63-lined polyubiquitin chain of TAK1 [19]. Recently, another TRAF family member TRAF4 has also been shown to trigger polyubiquitination of TAK1 and this modification is important for TAK1 activation [20]. Interestingly, a recent study demonstrated that TAB1 is modified with N-acetylglucosamine (so called *O*-GlcNAcylation) and this modification is required for full TAK1 activation [21].

TGF-β signaling plays an important role in the initiation, promotion and progression of many types of human cancer [22]. Because TAK1 is a key mediator of TGF-β signaling, it is expected that TAK1 may have a role in tumorigenesis. The involvement of TAK1 in breast cancer was firstly demonstrated by two groups. Safina et al identified TAK1 as an upstream activator of matrix metalloproteinase-9 (MMP-9) and is required for TGF-β-induced breast cancer metastasis [11]. Neil et al found a novel TAB1:TAK1:IκB kinase:NF-κB signaling axis that mediated the oncogenic activity of TGF-β in breast cancer [23]. In addition to metastasis promotion, TAK1 signaling increases resistance of breast cancer cells to paclitaxel and topoisomerase inhibitors [24, 25].

We previously found that TAK1 is constitutively activated in highly metastatic MDA-MB-231 breast cancer cells while its activity is very low in non-metastatic MCF-7 cells [26]. We also demonstrated that activation of TAK1 up-regulates the expression of chemokine (C-C motif) receptor 7 (CCR7) and increases the lymphatic invasion ability of breast cancer cells because lymphatic endothelial cells (LECs) express high level of CCR7 ligands CCL19 and CCL21 and induce a chemotactic effect on CCR7-expressing cancer cells. Collectively, our results suggest CCR7 as a critical target of TAK1 in the mediation of breast cancer metastasis. In this study, we extend our work to investigate the expression and association of TAK1 and CCR7 in breast tumor tissues. In addition, we tried to inhibit TAK1 by chemical inhibitors and siRNA to validate whether TAK1 is a therapeutic target of breast cancer.

## RESULTS

### CCR7 expression is associated with TAK1 activation

**Figure 1:**
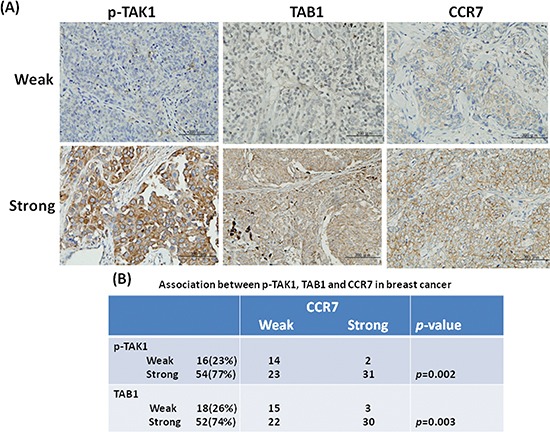
Association between p-TAK1, TAB1 and CCR7 in breast tumors **(A)** Immunohistochemical staining of breast tumors with weak or strong expression. **(B)** Association between p-TAK1 or TAB1 with CCR7 in a panel of tumor tissues from 70 patients.

Our previous study demonstrated that activation of TAK1 increases CCR7 expression in breast cancer cells [26]. To validate this association in primary tumor tissues, we investigated the expression of activated TAK1 by using an antibody which detected the phosphorylated Thr-178 and Thr-184 at the kinase activation loop of TAK1. As shown in Figures 1A and 1B, activated TAK1 was stained in the cytoplasm of cancer cells and was highly expressed in 77% (54/70) of the tumor tissues. Expression of CCR7 was appeared at the plasma membrane and was detected in 47% (33/70) of the cases. We found a strong association between activated TAK1 and CCR7 (*p* = 0.002) confirming our previous cell-based results. We also examined the expression of the TAK1 binding partner TAB1 and found the up-regulation of TAB1 in 74% of the tumor tissues. In addition, expression of TAB1 is significantly associated with CCR7 (*p* = 0.003).

### Inhibition of TAK1 reduces CCR7 expression

**Figure 2:**
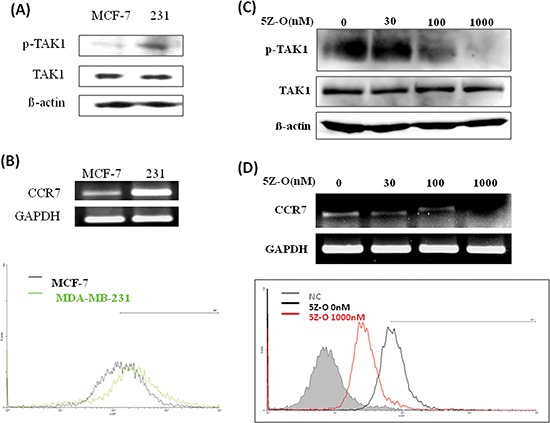
Inhibition of TAK1 attenuates CCR7 expression **(A)** Expression of phospho-TAK1 and TAK1 in MCF-7 and MDA-MB-231 (231) breast cancer cells. **(B)** Expression of CCR7 mRNA and cell surface CCR7 protein in MCF-7 and MDA-MB-231 (231) cells. **(C)** MDA-MB-231 cells were treated with different doses of TAK1 inhibitor 5Z-O for 24 h. TAK1 activity was examined by detecting p-TAK1 by immunoblotting. β-actin was used as an internal control. **(D)** MDA-MB-231 cells were treated with different doses of TAK1 inhibitor 5Z-O for 24 h. CCR7 mRNA was detected by RT-PCR and the cell surface CCR7 protein was studied by flow cytometry.

We next addressed whether TAK1 is an upstream regulator of CCR7. Our data showed that TAK1 activity is high in MDA-MB-231 cells while it is low in MCF-7 cells (Figure 2A). However, the protein level of TAK1 was similar in both cell lines indicating the increase of kinase activity was caused by upstream signaling stimulation. In consistent with our previous data, CCR7 is highly expressed in MDA-MB-231 cells and is low in MCF-7 cells (Figure 2B). Treatment of MDA-MB-231 cells with a selective TAK1 inhibitor 5Z-O dose-dependently inhibited TAK1 activity without affecting the expression of TAK1 protein (Figure 2C). In agreement with our hypothesis, 5Z-O reduced CCR7 mRNA in a dose-dependent manner. Flow cytometric analysis also demonstrated the reduction of CCR7 protein on cell surface by 5Z-O (Figure 2D). These effects were not due to the growth-inhibitory or cytotoxic activity of 5Z-O because the proliferation of MDA-MB-231 cells was only marginally reduced by 10–15% at the highest concentration (1000 nM) used in these experiments.

### TAK1 inhibitor 5Z-O suppresses downstream signaling and CCR7 promoter activity

**Figure 3:**
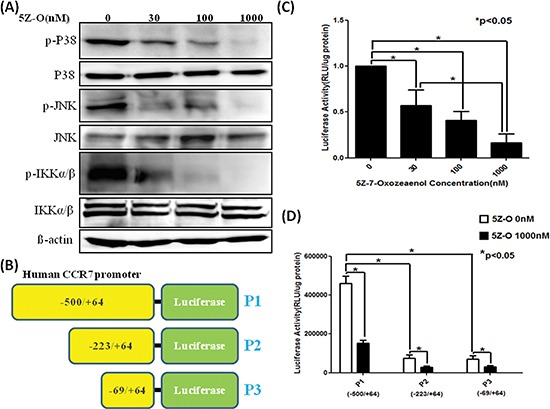
Inhibition of downstream signaling and CCR7 promoter by TAK1 inhibitor **(A)** MDA-MB-231 cells were treated with different doses of 5Z-O for 24 h. The kinase activity of p38, JNK and IKK was assayed by using phosphor-specific antibodies. **(B)** Scheme of the CCR7 promoter-luciferase plasmids P1, P2 and P3 used in reporter assays. **(C)** P1 plasmid was transfected into MDA-MB-231 cells by using Lipofectamine reagent. After transfection, cells were treated with vehicle (0.1% DMSO) or various concentrations of 5Z-O in 10% FCS medium for 24 h and luciferase activity was determined. The luciferase activity of the control group was defined as 1. *p < 0.05. **(D)** MDA-MB-231 cells were transfected with P1, P2 or P3 vectors and were incubated without or with 5Z-O for 24 h. Luciferase activity was measured and the difference between different experimental groups was compared. *p < 0.05.

The effect of 5Z-O on the activation of TAK1 downstream signaling was investigated. As shown in Figure 3A, the enzymatic activity of p38, JNK and IKK determined by detecting the phosphorylation status of these kinases was dose-dependently inhibited by 5Z-O. To ascertain whether TAK1 increases CCR7 via transcriptional activation, we cloned the promoter region of human CCR7 gene and created the P1 promoter-luciferase construct (−500/+64) (Figure 3B). Two additional deletion constructs P2 (−223/+64) and P3 (−69/+64) were generated from P1 plasmid. Our data demonstrated that 5Z-O inhibited the P1 promoter activity dose-dependently (Figure 3C). Although 5Z-O also inhibited the P2 and P3 promoter activity, we thought the major responsive elements by which TAK1 regulates CCR7 expression is located within the −500 to −223 region because deletion of this sequence caused a 80% of reduction of promoter activity in TAK1-activated MDA-MB-231 cells (Figure 3D).

### NF-κB and c-JUN are important for TAK1-increased CCR7 expression

**Figure 4:**
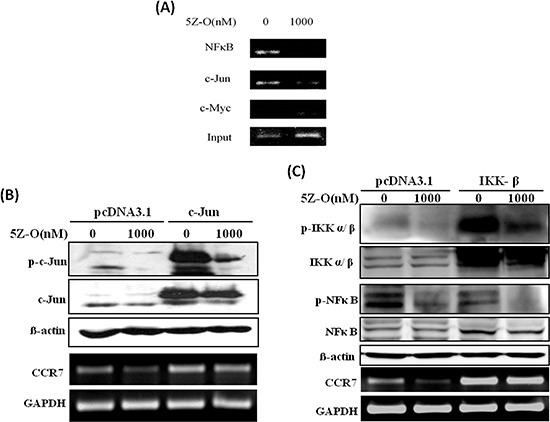
NF-κB and c-JUN are important for TAK1-increased CCR7 expression **(A)** MDA-MB-231 cells were treated without or with 5Z-O (1000 nM) for 24 h. Genomic DNA was isolated and the binding of NF-κB and c-JUN to CCR7 promoter was investigated by ChIP assay. Anti-Myc antibody was used as a negative control to confirm the specific binding of NF-κB and c-JUN. **(B)** MDA-MB-231 cells were transfected with pcDNA3.1 or c-Jun expression vector. After 48 h, cells were treated without or with 5Z-O (1000 nM) for another 24 h. c-JUN and p-c-JUN were detected by immunoblotting and β-actin was used as internal control. CCR7 mRNA expression was investigated by RT-PCR and GAPDH was used as internal control. **(C)** MDA-MB-231 cells were transfected with pcDNA3.1 or IKK-β expression vector. After 48 h, cells were treated without or with 5Z-O (1000 nM) for another 24 h. IKK, p-IKK, NF-κB and p-NF-κB were studied by immunoblotting and β-actin was used as internal control. CCR7 mRNA expression was investigated by RT-PCR and GAPDH was used as internal control.

We next addressed the potential transcription factors involved in the regulation of CCR7 by TAK1. Two lines of evidence led us to investigate NF-κB and AP-1. First, a previous study demonstrated that these two transcription factors are important for in the regulation of CCR7 expression in metastatic squamous cell carcinoma of head and neck [27]. Second, our current data showed that TAK1 activated IKK and JNK, two upstream kinases which play critical roles in the activation of NF-κB and AP-1. ChIP assays indicated that NF-κB and c-JUN are constitutively bound to human CCR7 promoter and their binding is significantly inhibited by 5Z-O (Figure 4A). Ectopic expression of c-JUN dramatically induced c-JUN activation (as demonstrated by increase of p-c-JUN) and rescued the down-regulation of CCR7 by 5Z-O (Figure 4B). In addition, overexpression of IKK-β enhanced NF-κB activation (as demonstrated by phosphorylation of Ser276 which increases interaction with the transcriptional coactivator p300/CBP to enhance NF-κB transcriptional activity) also effectively counteracted 5Z-O-induced inhibition (Figure 4C).

### Knockdown of TAB1 reduces tumor growth, lymph node invasion and lung metastasis

**Figure 5:**
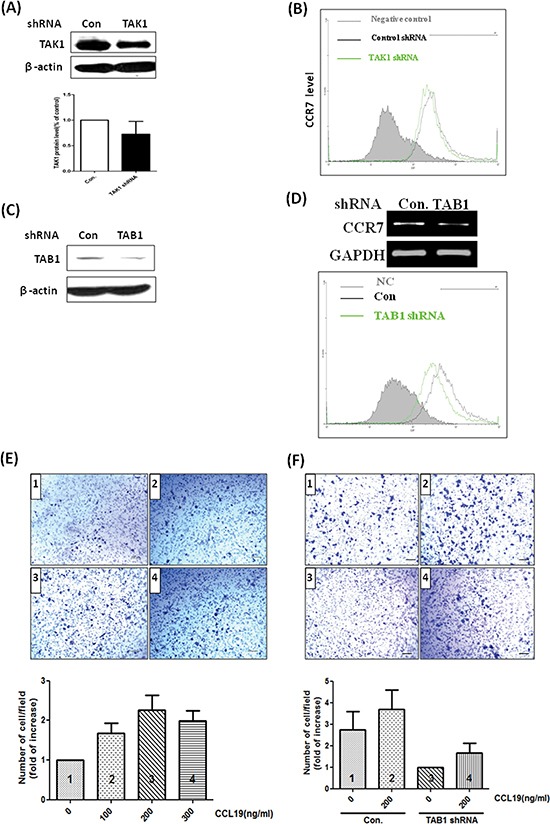
Knockdown of TAB1 reduces CCR7 expression and CCL19-induced chemotaxis of breast cancer cells **(A)** MDA-MB-231 cells were transfected with control luciferase shRNA (Con) or TAK1 shRNA and selected by puromycin (5 μg/ml) for 3 weeks to generate stable clone. TAK1 protein level was studied by immunoblotting. **(B)** Cell surface CCR7 protein of control and TAK1-inhibited clones were investigated by flow cytometry. **(C)** MDA-MB-231 cells were transfected with control luciferase shRNA (Con) or TAB1 shRNA and selected by puromycin (5μg/ml) for 3 weeks to generate stable clone. TAB1 protein level was studied by immunoblotting. **(D)** CCR7 mRNA was studied by RT-PCR and cell surface CCR7 protein of control and TAB1-inhibited clones were investigated by flow cytometry. **(E)** MDA-MB-231 cells were cultured on the upper unit of the Transwell system and serum-free medium containing different concentrations of CCL19 was placed in the lower unit. After 24 h, cells migrated to the opposite side of the Transwell membrane were counted and the results were expressed as fold of increase of chemotaxis compared to the control group (without CCL19 stimulation). **(F)** Control or TAB1-inhibited clones were subjected to chemotaxis assay as described above. The number of migratory cells of the TAB1-inhibited cells cultured with serum-free and CCL19-free medium was defined as 1. The results of other experimental groups were compared with this group.

**Figure 6:**
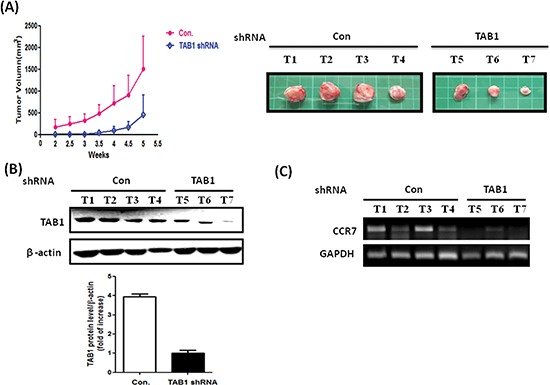
Reduction of *in vivo* tumor growth in TAB1-inhibited MDA-MB-231 cells **(A)** Control or TAB1 knockdown stable clones were injected into left second thoracic mammary fat pad of nude mice and tumor volumes were measured every 3 days from the second week after injection. Mice were sacrificed at week 5 after cell injection and tumors were removed. Cellular protein and mRNA were isolated from tumors for analysis. **(B)** TAB1 protein expression in control (Con) and TAB1-inhibited tumors was investigated by immunoblotting. TAB1 protein level was normalized to β-actin and the averaged level of TAB1-inhibited group was defined as 1. The TAB1 protein was about 4-fold higher than that of TAB1-inhibited group. **(C)** CCR7 mRNA of tumors of control (Con) and TAB1-inhibited groups was compared. A significant reduction of CCR7 was observed in TAB1-inhibited group.

To clarify the role of TAK1 in breast cancer *in vivo*, we established TAK1 and TAB1 knockdown stable clones by using short-hairpin RNA (shRNA) methodology. As shown in Figure 5A, the abundance of TAK1 is high in MDA-MB-231 cells and was only marginally reduced by shRNA. In the clone, expression of CCR7 was not significantly affected (Figure 5B) indicating targeting of TAK1 may not be a good strategy. Conversely, knockdown of TAB1 in MDA-MB-231 cells effectively reduced TAB1 protein (Figure 5C). In addition, the mRNA and protein level of CCR7 were significantly reduced in the clone (Figure 5D). We next tested the CCL19-induced chemotaxis on control and TAB1-inhibited clone. As shown in Figure 5E, CCL19 increased the chemotaxis of MDA-MB-231 cells in a dose-dependent manner with the maximal effect found at 200 ng/ml. Knockdown of TAB1 attenuated both basal and CCL19-induced chemotaxis (Figure 5F). Therefore, we used the TAB1-inhibited clone to do animal experiment. Indeed, tumor growth was reduced in TAB1-inhibited clone (Figure 6A). Analysis of TAB1 expression in tumor tissues confirmed that TAB1 protein was reduced by 70% in the knockdown clone when compared to that of control vector-transduced clone (Figure 6B). More importantly, CCR7 expression was significantly repressed in TAB1-inhibited clone suggesting the TAB1/TAK1 signaling controls CCR7 *in vivo* (Figure 6C).

**Figure 7:**
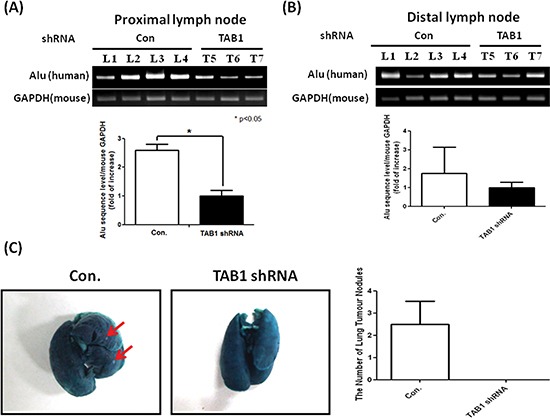
Inhibition of lymph node and lung metastasis by TAB1 knockdown **(A, B)** Total RNA was isolated from proximal and distal lymph nodes and expression of human Alu sequence which reflected the invasion of MDA-MB-231 cells into mouse lymph nodes was investigated by RT-PCR. The expression level of the TAB1-inhibited group was defined as 1. *p < 0.05. **(C)** Mouse lungs were stained with a contrast solution (15% India ink in distilled water) by injecting the solution into the trachea during animal sacrifice and then fixed in a fixation solution (100 mL of 70% alcohol, 10 mL of formaldehyde, and 5 mL of glacial acetic acid). Metastatic tumor nodules on the surface of the lungs were counted.

We next investigated whether inhibition of the TAB1/TAK1 signaling (which reduced CCR7 expression) affects tumor metastasis. Invasion of MDA-MB-231 cells into mouse axillary lymph nodes was examined by detecting the expression of human Alu sequence. Lymph nodes of the control group exhibited high level of Alu sequence while it was reduced by 60% in TAB1-inhibited group (Figure 7A). Similar results were also observed in distal lymph nodes that isolated from the counter side of tumor injection site (Figure 7B). In addition, metastatic nodules were found in the lungs of the control group while they were not detectable in TAB1-inhibited group (Figure 7C).

## DISCUSSION

Although TAK1 is known to involve in tumorigenesis via induction of a number of downstream target genes, the activation status (enzymatic activity) of TAK1 in primary tumor tissues and its association with target gene expression have little been investigated. In this study, we demonstrate for the first time that TAK1 is highly activated in human breast tumor tissues by detecting the phospho-TAK1 level. In addition, we provide clear evidence that expression of activated TAK1 is strongly associated with the up-regulation of CCR7 in tumor tissues confirming our previous results that TAK1 activation increases CCR7 expression in cultured breast cancer cells. Moreover, we extend our study to show that inhibition of TAK1 by knockdown of its binding protein TAB1 leads to attenuation of CCR7 expression and reduction of lymph node invasion and distant lung metastasis. We also elucidate the mechanism by which TAK1 controls CCR7 expression and find that TAK1 activates IKK and JNK kinases to increase the binding of NF-κB and c-JUN to CCR7 promoter to stimulate its transcription. These results support an oncogenic role of TAK1 in human breast cancer.

We also found that knockdown of TAB1 reduces migration and invasion of MDA-MB-231 cells (data not shown). In consistent with previous study, our data showed the expression of MMP-2 and MMP-9 is inhibited in TAB1-inhibited cells (Supplementary Figures 1A and 1B). Interestingly, we found that the cell-cell junction protein E-cadherin is re-expressed after knockdown of TAB1. E-cadherin is a master epithelial cell marker and is a key regulator of epithelial-mesenchymal transition (EMT) which expression is tightly controlled by several transcription repressors. Our data demonstrated that expression of Zeb1 and Zeb2 but not Twist and Slug are repressed by TAB1 inhibition indicating Zeb1 and Zeb2 may be involved in the suppression of E-cadherin by TAK1. Because TAK1 was originally identified as a kinase activated by TGF-β, an important EMT promoter in diverse cancer types, it is not surprising that inhibition of TAK1 activity could reverse the EMT phenotype and re-activate E-cadherin expression. Recently, Lam et al demonstrated Zeb1 and Snail are altered in TAK1-deficient metastatic skin cancer cells [28]. How TAK1 modulates the expression of transcription repressors to control E-cadherin expression and EMT is still unknown. In addition, the effect of TAK1 on the transcription repressors may be cell-type dependent because we did not see the alteration of Snail and Slug in our TAB1-inhibited breast cancer cells. Therefore, the regulation of EMT by TAK1 is complex and needs to be elucidated in specific cancer types. Taken together, we suggest a signaling network activated by TAK1 to promote invasion and metastasis in breast cancer (Supplementary Figure 2).

In addition to promotion of metastasis, TAK1 plays a critical role in therapeutic resistance. This is largely due to the activation of NF-κB and AP-1 by TAK1 which suppresses proapototic signaling pathways and reduces the sensitivity of cancer cells to chemotherapy or radiotherapy. In pancreatic cancer, inhibition of TAK1 by RNAi knockdown or an oral inhibitor LYTAK1 significantly enhanced the cytotoxic effect of oxaliplatin and gemcitabine [29]. In nude mice, administration of LYTAK1 and gemcitabine reduced tumor burden and prolonged animal survival. In neuroblastoma, 5Z-O, the TAK1 inhibitor used in this study, augmented the cell-killing activity of doxorubicin and etoposide [30]. Recently, Zhao et al showed that a tumor suppressor miRNA miR-26b inhibits cancer growth by targeting TAK1 and TAB3 [31]. Ectopic expression of this miRNA enhances the chemosensitivity of hepatocellular carcinoma cells to TNF-α and doxorubicin. Beside chemoresistance, TAK1 also increases radioresistance. Han et al demonstrated that radiation induces TAK1 activation in MDA-MB-231 cells and this activation attenuates radiation-induced apoptosis by inducing autophagy [32]. Treatment of TAK1 or autophagy inhibitors overcomes radioresistance in cancer cells. These studies suggest TAK1 is a rational target for cancer treatment. Small molecules inhibitors of TAK1 have been described recently. 5Z-O, a resorcylic lactone isolated from fungal origin, is an irreversible ATP-competitive inhibitor of TAK1 [33]. Chemically synthesized oxindole derivates also show potent inhibitory effect on TAK1 at nanomolar to low micromolar concentration [34]. More recently, a series of 7-aminofuro[2,3-*c*]pyridine inhibitors of TAK1 have been described [35]. These inhibitors repressed proliferation of colon and ovarian cancer cells *in vitro* and reduced tumor growth in xenograft model. In this study, we showed that TAK1 is highly activated in breast cancer and inhibition of TAK1 significantly suppressed tumor growth and metastasis. Our results suggest TAK1 is a potential therapeutic target for breast cancer.

## MATERIALS AND METHODS

### Experimental materials

TAK1 kinase inhibitor, 5Z-7-Oxozeaenol (5Z-O) was purchased from Tocris Bioscience (catalog number 3604). Antibodies against TAK1 (catalog number 4505), phospho-TAK1(Thr184/187) (catalog number 4531), TAB1(catalog number 3225), p38 MAP Kinase(catalog number 9212), phospho-p38 MAP Kinase(Thr180/Tyr182) (catalog number 9211), JNK(catalog number 9252), phospho-JNK(Thr183/Tyr185) (catalog number 9251), phospho-c-Jun (Ser73) (catalog number 9164), phospho-IKKalpha/beta (Ser176/180) (catalog number 2687), phospho-NF-κB p65 (Ser276) (catalog number 3037) were obtained from Cell Signaling (Danvers, MA). Antibodies against c-Jun, IKKα/β, IκB-α, NFκB p65 were purchased from Santa Cruz Biotechnology (Santa Cruz, CA). Antibodies against human CCR7 (catalog number 2059-1) and TAB1 (catalog number 2139-1) for immunohistochemical analysis were obtained from Epitomics (Burlingame, CA). Anti-CCR7 (catalog number MAB197) antibody for flow cytometry was obtained from R&D Systems (Minneapolis, MN). Anti-phospho-TAK1 (Thr187) antibody for immunohistochemical analysis was obtained from Abgent (San Diego, CA). IKK-β expression vector was kindly provided by Dr. Michael Karin (University of California San Diego). c-Jun expression vector was provided by Dr. Ben-Kuen Chen (Department of Pharmacology, College of Medicine, and Center for Gene Regulation and Signal Transduction Research, National Cheng Kung University, Tainan, Taiwan). CCR7 promoter luciferase reporter plasmids P1, P2 and P3 were constructed from genomic DNA by PCR amplification as described previously [36].

### Cell culture

MCF-7 and MDA-MB-231 breast cancer cells were obtained from the cell bank of the National Health Research Institute (Miaoli, Taiwan). MCF-7 cell line was cultured in Dulbecco's modified Eagle's medium/F-12 medium and MDAMB-231 was in RPMI 1640 medium, both containing 10% charcoal-stripped fetal calf serum (FCS) and antibiotics.

### Reverse transcription-polymerase chain reaction (RT-PCR)

Total RNA was subjected to RT-PCR analysis as described previously. The primer sequences: CCR7-forward: 5′-GGACCTGGGGAAACCAAT-3′; CCR7-reverse: 5′-GCCAGGTTGAGCAGGTAGGT-3′; GAPDH-forward: 5′-GAGTCAACGGATTTGGTCGT-3′; GAPDH-reverse: 5′-TGTGGTCATGAGT CCTTCCA-3′; TAB1-forward: 5′-GGATCGGGGATTACAAGGTT-3′; TAB1-reverse: 5′-TGCTTGGCAAACTCAGTGTC-3′. Amplified cDNA products were run on agarose gels, stained with ethidium bromide, and visualized under UV light.

### Immunoblotting

Control- or drug-treated cells were washed with ice-cold phosphate-buffered saline and harvested in a lysis buffer as described previously [37]. Equal amounts of cellular proteins were resolved on 10% SDS-PAGE gels, transferred electrophoretically onto nitrocellulose membranes, blocked in 5% milk and the blots were incubated with different primary antibodies. The resulting immunocomplexes were visualized by enhanced chemiluminescence.

### Flow cytometry

For detection of CCR7 protein expression on the cell surface, cells were washed with ice-cold phosphate-buffered saline and incubated with biotinylated anti-CCR7 antibody at 4°C for 30 min. After washing, cells were incubated with fluorescein isothiocyanate-conjugated avidin and subjected to flow cytometric analysis as described previously [38].

### Promoter activity assays

MDA-MB-231 cells (2 × 10^5^ per well) were cultured on 6-well plates and were transfected with 2μg CCR7 promoter luciferase reporter plasmid P1 (−500/+64), P2 (−223/+64) or P3 (−69/+64). After transfection, cells were treated with vehicle (0.1% DMSO) or various concentrations of 5Z-O in 10% FCS medium for 24 h. Promoter activity was determined and normalized as described previously [24].

### Chromatin immunoprecipitation (ChIP) assay

Vehicle- or 5Z-O-treated cells were fixed with 1% formaldehyde at 37°C for 10min. Cells were washed twice with ice-cold phosphate-buffered saline containing protease inhibitors (1 mM phenylmethylsulfonyl fluoride, 1 μg/ml aprotinin, and 1 μg/ml pepstatin A), scraped, and pelleted by centrifugation at 4°C. Cells were resuspended in a lysis buffer (1% SDS, 10 mM EDTA, and 50 mM Tris-HCl, pH 8.1), incubated for 10 min on ice, and sonicated to shear DNA. After sonication, lysate was centrifuged for 10 min at 13,000 rpm at 4°C. The supernatant was diluted in ChIP dilution buffer (0.01% SDS, 1% Triton X-100, 2mM EDTA, 16.7 mM Tris-HCl, pH 8.1, 167 mM NaCl, and protease inhibitors). Anti-NF-κB (p65), anti-c-Jun or anti-Myc (negative control) antibodies were added to the supernatant and incubated overnight at 4°C with rotation. ChIP assays were performed as described previously [35]. DNA fragments were recovered and were subjected to PCR amplification by using the primers specific for the detection of regions which contained the NF-κB (−399/−391) and c-Jun (−320/−309) binding sites of human CCR7 gene promoter. The sequences for the primers: forward: 5′-CCTCCCATTGGCCTAAACTA-3′; reverse: 5′-CAGGCCATGGGTTAGATGTT-3′.

### shRNA targeting

shRNAs carrying puromycin selection marker were purchased from the National RNAi Core Facility of Academic Sinica (Taiwan). The sequences used for targeting were as follows: TAK1, 5′-GATCCGTAGATCCATCCAAGACTTTTCAAGAGA AAGTCTTGGATGGATCTACTTA-3′; TAB1, 5′-GATC CAGTCCTTCTCA ACAGCAAGTTCAAGAGACTTGC TGTTGAGAAGGACTGCA-3′. Cells were transfected with the shRNA plasmids and selected by puromycin (5μg/ml) for 3 weeks. The expression of TAK1 and TAB1 was investigated, and the stable clone with the highest knockdown efficiency was used for animal study.

### Animal study

Animal studies were approved by the Animal Care and Ethics Committee of the National Sun Yat-Sen University. Female BALB/cAnN-Foxn1 null mice (5 weeks old) were obtained from National Laboratory Animal Center (Taipei, Taiwan). Mice were randomly divided into two groups (*n* = 5 for each group) and subjected to treatment. The control and TAB1 knockdown MDA-MB-231 cells (1 × 10^6^ cells/mice) were injected into left second thoracic mammary fat pad of nude mice. Tumor volumes were measured every 3 days from the second week after injection and were calculated using the formula, *V* = (length) × (width)^2^ × 0.5. After 6 weeks, animals were sacrificed and the tumors were excised and subjected for immunoblotting and RT-PCR. Left axillary lymph nodes (defined as proximal lymph nodes because they located at the same side of tumor injection site) were excised and subjected for PCR to detect human Alu sequence by using the following primers: forward: 5′-CACCTGTAATCCCAGCACTTT-3′; reverse: 5′-CCC AGGCTGGAGTGCAGT-3′. In addition, right axillary lymph nodes (defined as distal lymph nodes because they located at the counter side of tumor injection site) were also excised for PCR analysis.

### Immunohistochemical analysis

Human breast cancer tissue array (catalog number BR8015) was purchased from US Biomax (Rockville, MD). The tumor samples included 58 invasive ductal carcinoma, 8 medullary carcinoma and 4 ductal-lobular mixed carcinoma and the array information was included in Supplementary Table 1. Tissues were placed in citrate buffer and incubated at 112°C for 20 min for antigen retrieval. Tissues were blocked with a 3% hydrogen peroxide solution to inhibit endogenous peroxidase activity and washed with phosphate-buffered saline. Tissues were probed with antibodies and bound primary antibodies were detected with horseradish peroxidase-conjugated secondary antibody and then developed by diaminobenzidine substrate. Finally, sections were co-stained with hematoxylin. The distribution of positive staining was graded as follows: 1: less than 1% of cells; 2: 1–10% of cells (weak staining); 3: 11–50% of cells and 4: >50% of cells. The intensity of positive staining was graded as 0: negative; 1: weak and 2: strong. The distribution and intensity scores were multiplied to obtain the final score. The score ≧ 6 was defined as strong staining.

### Statistical analysis

The associations of TAB1, p-TAK1 and CCR7 in breast tumor tissues were assessed using χ^2^ test and binary logistic regression. Student's *t* test was used to evaluate the difference between various experimental groups. Differences were considered to be significant at *p* < 0.05.

## SUPPLEMENTARY FIGURES AND TABLE


