# Supplementary material for: Targeting of TGF-β-activated protein kinase 1 inhibits chemokine (C-C motif) receptor 7 expression, tumor growth and metastasis in breast cancer
Source: Oncotarget. 2014 Dec 10;6(2):995–1007. doi: 10.18632/oncotarget.2739 (PMC4359270; doi:10.18632/oncotarget.2739)
Supplement: Supplementary file 1 [file oncotarget-06-995-s001.pdf]

SUPPLEMENTARY FIGURES AND TABLE

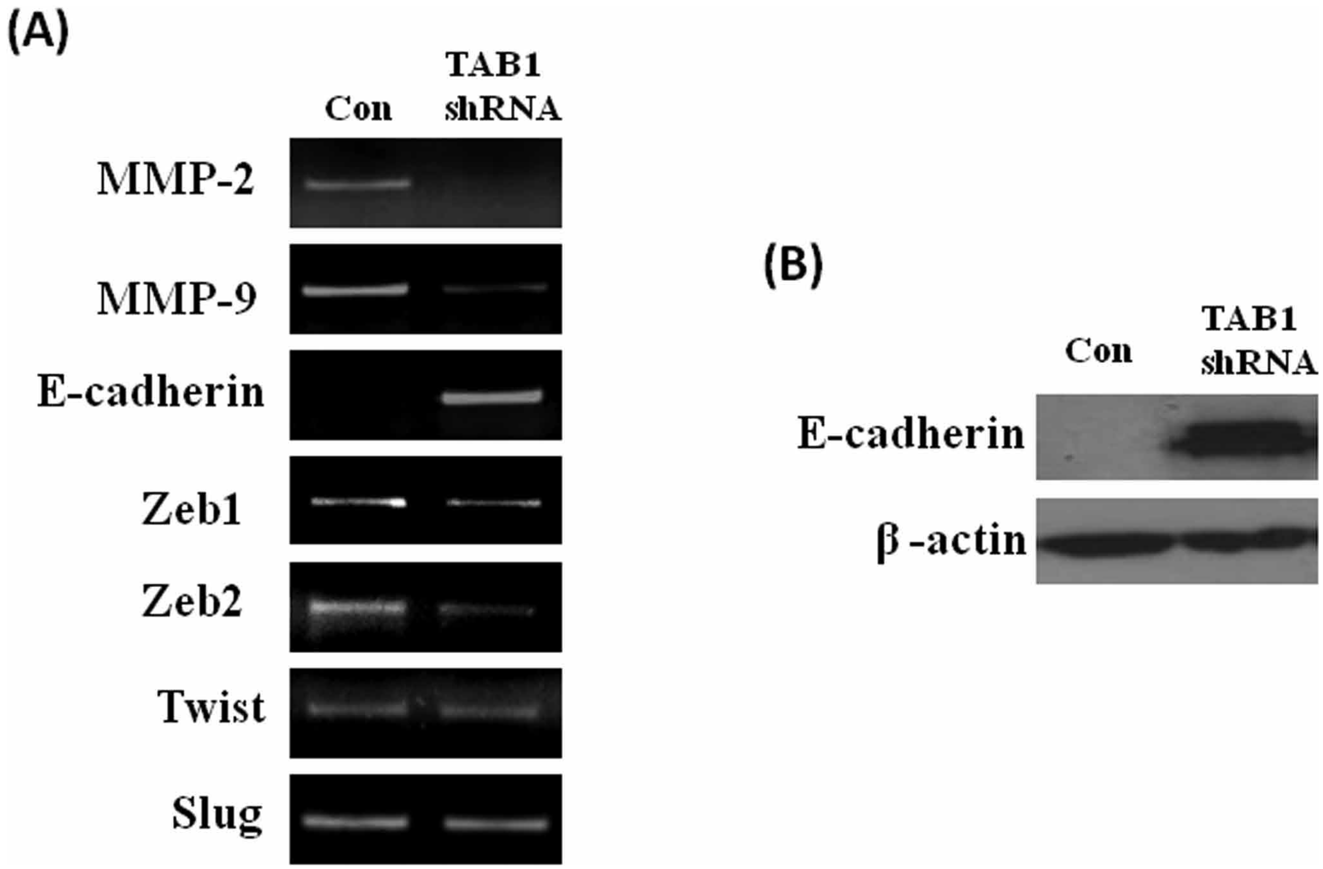

Supplementary Figure 1: Alteration of EMT regulators in TAB1-inhibited MDA-MB-231 cells.

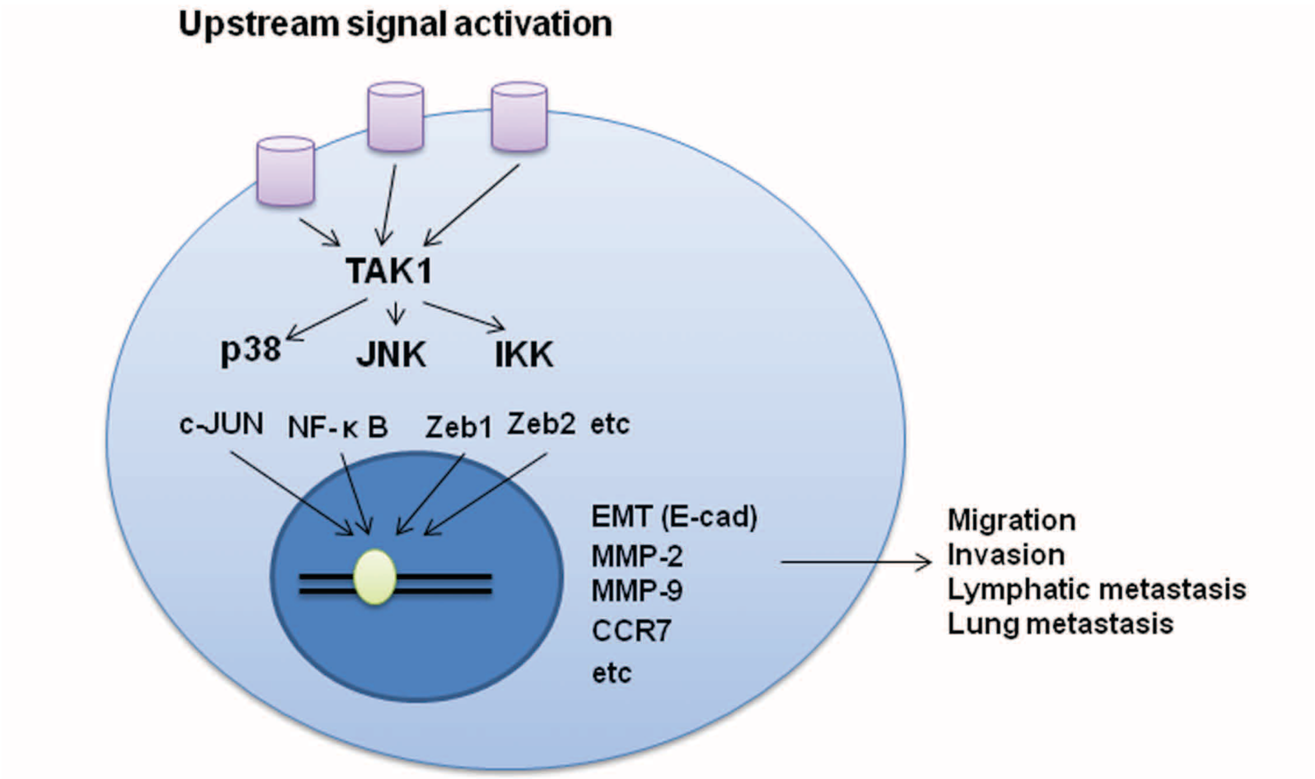

Supplementary Figure 2: Proposed mechanism of TAK 1-induced invasion and metastasis in breast cancer.

Supplemental Table 1: Microarray panel display

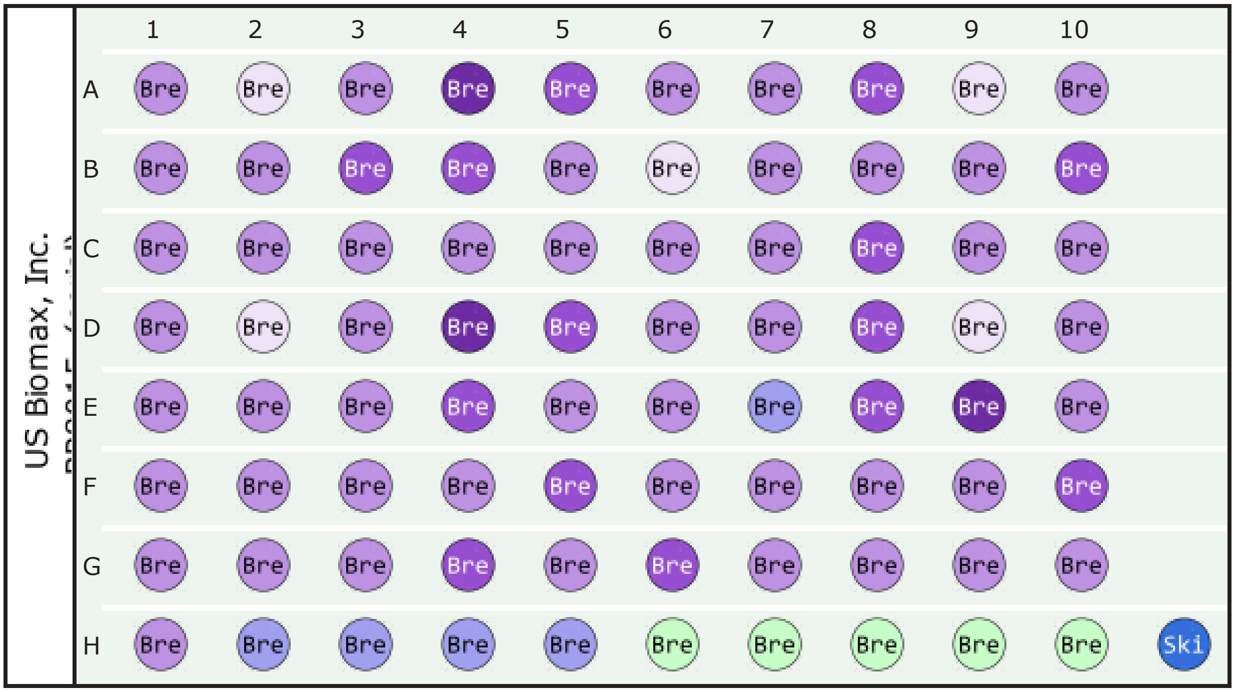

Legend: Bre - Breast

- Malignant tumor, - Malignant tumor (stage I), - Malignant tumor (stage IIIa), - Malignant tumor (stage IIIb), - Malignant tumor (stage IIa), - Malignant tumor (stage IIb), - Malignant tumor (stage IV), - NAT, - Normal tissue

Specification Sheet

Pos	No.	Sex	Age	Organ	Pathology diagnosis	Grade	Stage	TNM	Type †
A1	1	F	41	Breast	Invasive ductal carcinoma	2	IIb	T2N1M0	Malignant
A2	2	F	38	Breast	Invasive ductal carcinoma	1	I	T1cN0M0	Malignant
A3	3	F	55	Breast	Invasive ductal carcinoma	2	IIb	T3N0M0	Malignant
A4	4	F	62	Breast	Invasive ductal carcinoma	2	IV	T4N2M1	Malignant
A5	5	F	35	Breast	Invasive ductal carcinoma	2	IIIb	T4N2M0	Malignant
A6	6	F	41	Breast	Invasive ductal carcinoma	1	IIb	T3N0M0	Malignant
A7	7	F	75	Breast	Invasive ductal carcinoma	2	IIb	T2N1M0	Malignant
A8	8	F	43	Breast	Invasive ductal carcinoma (breast tissue)	–	IIIb	T4N2M0	Malignant
A9	9	F	32	Breast	Invasive ductal carcinoma	2	I	T1N0M0	Malignant
A10	10	F	47	Breast	Invasive ductal carcinoma	2	IIa	T2N0M0	Malignant
B1	11	F	60	Breast	Invasive ductal carcinoma	2	IIb	T2N1M0	Malignant
B2	12	F	53	Breast	Invasive ductal carcinoma	2	IIb	T3N0M0	Malignant
B3	13	F	48	Breast	Invasive ductal carcinoma	2	IIIb	T4N2M0	Malignant
B4	14	F	49	Breast	Invasive ductal carcinoma	2	IIIb	T4N1M0	Malignant
B5	15	F	40	Breast	Invasive ductal carcinoma	2	IIa	T2N0M0	Malignant
B6	16	F	45	Breast	Invasive ductal carcinoma	2	I	T1N0M0	Malignant
B7	17	F	51	Breast	Invasive ductal carcinoma	2	IIb	T3N1M0	Malignant
B8	18	F	44	Breast	Invasive ductal carcinoma	1	IIb	T2N1M0	Malignant
B9	19	F	53	Breast	Invasive ductal carcinoma	2	IIb	T2N1M0	Malignant
B10	20	F	42	Breast	Invasive ductal carcinoma	2	IIIb	T3N0M0	Malignant
C1	21	F	38	Breast	Invasive ductal carcinoma	2	IIa	T2N0M0	Malignant
C2	22	F	43	Breast	Invasive ductal carcinoma	2	IIb	T2N1M0	Malignant
C3	23	F	39	Breast	Invasive ductal carcinoma	2	IIb	T2N1M0	Malignant
C4	24	F	53	Breast	Invasive ductal carcinoma	2	IIa	T2N0M0	Malignant
C5	25	F	50	Breast	Invasive ductal carcinoma	3	IIb	T3N0M0	Malignant
C6	26	F	54	Breast	Invasive ductal carcinoma	2	IIb	T2N1M0	Malignant

(*Continued*)

Pos	No.	Sex	Age	Organ	Pathology diagnosis	Grade	Stage	TNM	Type †
C7	27	F	55	Breast	Invasive ductal carcinoma	1–2	IIb	T3N1M0	Malignant
C8	28	F	52	Breast	Invasive ductal carcinoma	2	IIIa	T2N2M0	Malignant
C9	29	F	32	Breast	Invasive ductal carcinoma	2	IIb	T2N1M0	Malignant
C10	30	F	50	Breast	Invasive ductal carcinoma	2	IIb	T2N1M0	Malignant
D1	31	F	50	Breast	Invasive ductal carcinoma	2	IIb	T2N1M0	Malignant
D2	32	F	53	Breast	Invasive ductal carcinoma	2	I	T1cN0M0	Malignant
D3	33	F	47	Breast	Invasive ductal carcinoma	2	IIb	T3N0M0	Malignant
D4	34	F	68	Breast	Invasive ductal carcinoma (sparse)	2	IV	T4N2M1	Malignant
D5	35	F	45	Breast	Invasive ductal carcinoma	2	IIIb	T4N2M0	Malignant
D6	36	F	40	Breast	Invasive ductal carcinoma	2	IIb	T3N0M0	Malignant
D7	37	F	47	Breast	Invasive ductal carcinoma	2	IIb	T2N1M0	Malignant
D8	38	F	57	Breast	Invasive ductal carcinoma	2	IIIb	T4N2M0	Malignant
D9	39	F	54	Breast	Invasive ductal carcinoma	2	I	T1N0M0	Malignant
D10	40	F	35	Breast	Invasive ductal carcinoma	1–2	IIa	T2N0M0	Malignant
E1	41	F	19	Breast	Invasive ductal carcinoma (sparse)	2	IIb	T2N1M0	Malignant
E2	42	F	28	Breast	Invasive ductal carcinoma	2	IIa	T1N1M0	Malignant
E3	43	F	38	Breast	Invasive ductal carcinoma	3	IIa	T2N0M0	Malignant
E4	44	F	65	Breast	Invasive ductal carcinoma	3	IIIa	T1N2M0	Malignant
E5	45	F	52	Breast	Invasive ductal carcinoma	3	IIb	T2N1M0	Malignant
E6	46	F	48	Breast	Invasive ductal carcinoma	3	IIb	T2N1M0	Malignant
E7	47	F	49	Breast	Cancer adjacent normal breast tissue	–	–	–	NAT
E8	48	F	41	Breast	Invasive ductal carcinoma	3	IIIa	T3N1M0	Malignant
E9	49	F	48	Breast	Invasive ductal carcinoma	3	IV	T2N1M1	Malignant
E10	50	F	44	Breast	Invasive ductal carcinoma	3	IIa	T2N0M0	Malignant
F1	51	F	59	Breast	Invasive ductal carcinoma	3	IIa	T2N0M0	Malignant
F2	52	F	61	Breast	Ductal-lobular mixed carcinoma	–	IIb	T2N1M0	Malignant
F3	53	F	38	Breast	Ductal-lobular mixed carcinoma	–	IIa	T2N0M0	Malignant
F4	54	F	46	Breast	Ductal-lobular	–	IIa	T2N0M0	Malignant

(*Continued*)

Pos	No.	Sex	Age	Organ	Pathology diagnosis	Grade	Stage	TNM	Type †
					mixed carcinoma				
F5	55	F	46	Breast	Ductal-lobular mixed carcinoma	–	IIIb	T4N0M0	Malignant
F6	56	F	46	Breast	Invasive lobular carcinoma (adenosis)	–	IIa	T2N0M0	Malignant
F7	57	F	49	Breast	Invasive lobular carcinoma	–	IIa	T2N0M0	Malignant
F8	58	F	38	Breast	Invasive lobular carcinoma (sparse)	–	IIb	T2N1M0	Malignant
F9	59	F	34	Breast	Invasive lobular carcinoma	–	IIa	T2N0M0	Malignant
F10	60	F	41	Breast	Invasive lobular carcinoma	–	IIIa	T2N2M0	Malignant
G1	61	F	43	Breast	Invasive lobular carcinoma	–	IIa	T2N0M0	Malignant
G2	62	F	56	Breast	Invasive lobular carcinoma	–	IIa	T2N0M0	Malignant
G3	63	F	40	Breast	Invasive lobular carcinoma	–	IIb	T2N1M0	Malignant
G4	64	F	40	Breast	Medullary carcinoma	–	IIIa	T2N2NM0	Malignant
G5	65	F	43	Breast	Medullary carcinoma	–	IIa	T2N0M0	Malignant
G6	66	F	48	Breast	Medullary carcinoma	–	IIIb	T4N0M0	Malignant
G7	67	F	31	Breast	Medullary carcinoma	–	IIa	T2N0M0	Malignant
G8	68	F	72	Breast	Medullary carcinoma	–	IIa	T2N0M0	Malignant
G9	69	F	40	Breast	Medullary carcinoma	–	IIa	T2N0M0	Malignant
G10	70	F	47	Breast	Medullary carcinoma	–	IIa	T2N0M0	Malignant
H1	71	F	55	Breast	Medullary carcinoma	–	IIa	T2N0M0	Malignant
H2	72	F	47	Breast	Cancer adjacent normal breast tissue	–	–	–	NAT
H3	73	F	43	Breast	Cancer adjacent normal breast tissue	–	–	–	NAT
H4	74	F	42	Breast	Cancer adjacent normal breast tissue	–	–	–	NAT
H5	75	F	53	Breast	Cancer adjacent normal breast tissue	–	–	–	NAT
H6	76	F	18	Breast	Normal breast tissue (fibrofatty tissue)	–	–	–	Normal
H7	77	F	19	Breast	Normal breast tissue	–	–	–	Normal
H8	78	F	35	Breast	Normal breast tissue	–	–	–	Normal
H9	79	F	50	Breast	Normal breast tissue	–	–	–	Normal
H10	80	F	27	Breast	Normal breast tissue	–	–	–	Normal

(Continued)

Pos	No.	Sex	Age	Organ	Pathology diagnosis	Grade	Stage	TNM	Type †
–	–	M	58	Skin	Malignant melanoma (tissue marker)	–			Malignant

**The grade 1-3 (or I-III) in Pathology Diagnosis is equivalent to well-differentiated, moderately-differentiated or poorly differentiated, respectively, under microscope.
Grade 1 or well-differentiated: Cells appear normal and are not growing rapidly.
Grade 2 or moderately-differentiated: Cells appear slightly different than normal.
Grade 3 or poorly differentiated: Cells appear abnormal and tend to grow and spread more aggressively.
Grade 4 or undifferentiated: *(for certain tumors), features are not significantly distinguishing to make it look any different from undifferentiated cancers which occur in other organs.

TNM grading:

T - Primary tumor
Tx - Primary tumor cannot be assessed
T0 - No evidence of primary tumor
Tis - Carcinoma in situ; intraepithelial or invasion of lamina propria
T1 - Tumor invades submucosa
T2 - Tumor invades muscularis propria
T3 - Tumor invades through muscularis propria into subserosa or into non-peritonealized pericolic or perirectal tissues.
T4 - Tumor directly invades other organs or structures and/or perforate visceral peritoneum

N - Regional lymph nodes
Nx - Regional lymph nodes cannot be assessed
N0 - No regional lymph node metastasis
N1 - Metastasis in 1 to 3 regional lymph nodes
N2 - Metastasis in 4 or more regional lymph nodes

M - Distant metastasis
Mx - Distant metastasis cannot be assessed
M0 - No distant metastasis
M1 - Distant metastasis
